# Bi-Directional SIFT Predicts a Subset of Activating Mutations

**DOI:** 10.1371/journal.pone.0008311

**Published:** 2009-12-14

**Authors:** William Lee, Yan Zhang, Kiran Mukhyala, Robert A. Lazarus, Zemin Zhang

**Affiliations:** 1 Department of Bioinformatics, Genentech, Inc., South San Francisco, California, United States of America; 2 Department of Protein Engineering, Genentech, Inc., South San Francisco, California, United States of America; Washington University, United States of America

## Abstract

Advancements in sequencing technologies have empowered recent efforts to identify polymorphisms and mutations on a global scale. The large number of variations and mutations found in these projects requires high-throughput tools to identify those that are most likely to have an impact on function. Numerous computational tools exist for predicting which mutations are likely to be functional, but none that specifically attempt to identify mutations that result in hyperactivation or gain-of-function. Here we present a modified version of the SIFT (Sorting Intolerant from Tolerant) algorithm that utilizes protein sequence alignments with homologous sequences to identify functional mutations based on evolutionary fitness. We show that this bi-directional SIFT (B-SIFT) is capable of identifying experimentally verified activating mutants from multiple datasets. B-SIFT analysis of large-scale cancer genotyping data identified potential activating mutations, some of which we have provided detailed structural evidence to support. B-SIFT could prove to be a valuable tool for efforts in protein engineering as well as in identification of functional mutations in cancer.

## Introduction

The growing amount of mutation and polymorphism data being generated has created a need for computational tools to systematically analyze large sets of mutations and filter them for those that have the greatest potential functional impact. Several sets of tools have become available that attempt to predict the functional impact of amino acid substitutions, thus providing a valuable arsenal for identifying mutations that should be the subject of further investigations [1]–[6]. The SIFT (Sorting Intolerant from Tolerant) algorithm [3], is arguably the most commonly used tool for detecting deleterious amino acid substitutions due to its easy application towards large numbers of mutations. However, SIFT and other tools like it only attempt to distinguish between two classes of mutations, often categorized as deleterious and tolerated [3] or non-neutral and neutral [6]. It has been shown that many important mutations, in cancer for example, are a result of activating or gain-of-function mutations. Most current tools do not make an effort to specifically identify such mutations and distinguish them from functionally deleterious substitutions. We hypothesize that there are at least three categories of activating mutations: mutations that destabilize the inactive form of a molecule thereby resulting in constitutive activation (e.g. EGFR L858R), mutations that mimic the activated state (e.g. phosphorylated) of a protein (e.g. BRAF V600E), and mutations that introduce an evolutionarily more common residue which enhances proteins activities. Our focus is on the latter form of activating mutations. These mutations may simply increase enzymatic activity or substrate binding through more beneficial biochemical interactions.

**Figure 1:**
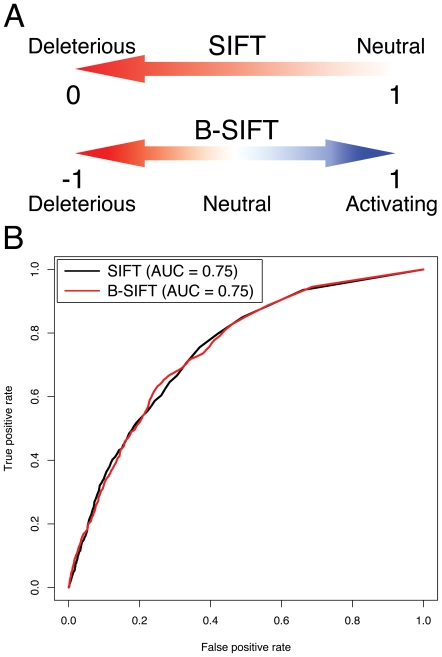
B-SIFT schematic and performance compared to SIFT. A. Schematic of B-SIFT scoring range versus original SIFT. SIFT generates scores for each substitution on a scale from 0 to 1, with scores closer to zero representing the mutations most likely to be deleterious. B-SIFT is bi-directional and takes the difference of SIFT scores between the wild-type and mutant alleles to obtain a score ranging from −1 to 1 with higher scores representing substitutions more likely to be activating mutations. B. Performance of B-SIFT versus SIFT in predicting deleterious mutations. A receiver-operator characteristic (ROC) plot showing the true positive versus false positive performance rates for B-SIFT (red curve, area under curve = 0.75) and SIFT (black curve, area under curve = 0.75) in predicting which of 4041 mutants of the *E. coli* LacI repressor gene are likely to have a deleterious functional impact [27], [29].

Here we present a modified version of SIFT called Bi-directional SIFT (B-SIFT) which is able to identify both deleterious and a subset of activating mutations given a protein sequence and a query mutation within that sequence. The SIFT algorithm relies upon evolutionary conservation to find mutations that have the greatest potential for negative functional impact and B-SIFT uses the same idea to find mutations with increased fitness. Intuitively, the concept is that mutating from an evolutionarily uncommon allele to one that is more commonly present in protein homologues could result in optimized protein activity. Rather than simply scoring the mutant allele based on the multiple protein sequence alignment, as SIFT does, B-SIFT calculates scores for both the mutant allele and the wild-type allele and returns the difference of these values as the final score, which effectively measures relative functional activity (Fig. 1A). In contrast to the two-category scoring that most bioinformatics tools output, B-SIFT scores can be interpreted with three categories such that low scores represent a deleterious effect, scores near zero represent a neutral effect, and high positive scores identify potential activating mutations.

To quantify B-SIFT's ability to classify mutations, we have validated B-SIFT against two protein mutation datasets: a diverse set of experimentally described mutagenesis experiments as curated in the SWISS-PROT protein database (MUTAGEN field [7]) and a large set of single amino acid substitution mutants in human DNase I. We find that high B-SIFT scores can effectively enrich for activating mutations in both datasets. The DNase I results demonstrate that B-SIFT could be capable of providing a starting point in protein engineering efforts by identifying candidate mutations for any protein, even one with minimal available structure or functional data (see Results S1 and Figure S1).

Perhaps the most important recent application of mutation analysis tools is in the realm of cancer research, where an influx of data regarding somatic mutations found in cancer emphasizes the need for efficient and reliable analysis methods [8]–[14]. Because of the inherent genetic instability of many cancers, it is known that many mutations found in cancer cells are a result of the cancer itself (passengers) rather than actual contributors to disease progression (drivers) [15].We have analyzed a large set of experimentally discovered cancer-associated somatic mutations with B-SIFT and performed a detailed structural analysis to predict the mutations most likely to be activating and potentially cancer-causing.

Hyperactive or gain-of-function mutations comprise an area of functional analysis that is often overlooked in large-scale mutation analyses. B-SIFT presents the first generalized tool for systematic prediction of potentially activating missense mutations that are a result of increased protein fitness, thereby identifying potentially functional mutations that were previously ignored. We show that B-SIFT can be used for identification of potential activating mutations while maintaining SIFT's ability to identify neutral and deleterious mutations.

## Materials and Methods

The original SIFT software (version 2.1.2) was downloaded from the official SIFT website (http://blocks.fhcrc.org/sift/SIFT.html). Protein sequences were retrieved from Uniprot for the siftalign program. Wrapper scripts were written to streamline protein sequence retrieval, cache alignment results, and enable batch processing of the input. B-SIFT score can be calculated as SIFT(mutant)–SIFT(wild-type) where SIFT(mutant) refers to the SIFT score calculated for the mutant allele and SIFT(wild-type) is the score calculated for the wild-type allele. According to SIFT documentation, results that have a median sequence information of greater than 3.25 are considered low confidence, so these results have been filtered from our analysis [16], [17].

SWISS-PROT mutagenesis data was downloaded and parsed from SWISS-PROT release 56. The MUTAGEN field from each protein entry was parsed out and merged into a single file containing all MUTAGEN entries. Each mutation was labeled as deleterious, activating, or neutral based on keyword recognition within only the first two words of the text description to prevent complications with exceptionally verbose phenotype descriptions. Mutations with descriptions that did not match any of the given strings were discarded from further analysis. Activating mutations contained at least one of: increase, enhance, activat, constitutive acti, restore. Mutations annotated as having a small or no effect were labeled as neutral, these were identified with keywords no effect, no change, normal, mild, minimal effect, minor, small effect, or wild-type. Deleterious mutations made up the majority of the dataset and also contained the greatest number of keywords: decrease, inhibit, reduc, loss, lower, abolish, abrogate, inactive, diminish, disrupt, impair, eliminate, no activity, prevent, suppress, increases km, increases the km. Mutations annotated as activating were then investigated by hand to remove any false positives, such as mutations described as “Increases substrate binding and reduces catalytic activity” or “Increases electrophoretic mobility of the protein.” This resulted in the removal of 104 out of 512 mutations (∼20%). The final dataset used for analysis included 408 activating, 1932 neutral, and 9736 deleterious mutations. The complete dataset, including B-SIFT scores and annotations, is available in Supplemental Data.

DNase I mutations were generated by site-directed mutagenesis and proteins were expressed in HEK293 cells using methods as previously described [18]–[20]. The methyl green assay was used to measure DNA hydrolytic activity of DNase I in the presence of 2 mM Mg^2+^ and 2 mM Ca^2+^ as reported previously [20], [21]. DNase I concentrations were determined by ELISA, using a goat anti-DNase I polyclonal antibody coat and detecting with a rabbit anti-DNase I polyclonal antibody conjugated to horseradish peroxidase as described previously [20], [21]. In both assays, multiple sample dilutions were compared to standard curves of wildtype DNase I to determine concentrations. The relative specific activity (RSA) was calculated by normalizing the specific activity of the mutant to the specific activity of wild-type DNase I. Our analyses of DNase I mutations are described in Results S1 and Figure S1.

SNP data was downloaded from NCBI dbSNP database build 126 (http://www.ncbi.nlm.nih.gov/projects/SNP/) [22]. Because B-SIFT uses an amino acid substitution as input and interpretation of allele frequencies would be complicated by multi-allelic SNPs, only bi-allelic missense SNPs were used in this analysis, resulting in a set of 32261 nonsynonymous SNPs (22,219 with median sequence information less than or equal to 3.25). Each nonsynonymous SNP was translated into the appropriate amino acid change for use as input into B-SIFT. Allele frequencies were determined from the SNPAlleleFreq.bcp file from the dbSNP FTP download site. The raw data used in our dbSNP analysis is available in Supplemental Data.

Cancer mutation data was obtained from the Sanger Institute Catalogue Of Somatic Mutations In Cancer web site (http://www.sanger.ac.uk/cosmic [23], [24]). Public cancer genome mutation data was downloaded from their respective publication sites [8], [9], [11], [12], [14] and run through B-SIFT with protein sequences corresponding to the given transcript identifiers in each publication. Mutations chosen for further structural analysis had either B-SIFT score greater than 0.5 or both a positive B-SIFT score and cancer-specific overexpression with a one-tailed t-test p-value less than 0.001. Expression data is extracted from the Gene Logic database (Gene Logic, Inc., Gaithersburg, MD, USA) and based on the average expression in cancer samples versus normal samples for the tissue that the mutation was found in. Expression differences are calculated as a p-value using a two-sample t-test for the average expression between cancer samples and normal samples.

Homology models were built using Modeler 9v4. Models of Pirh2 A190 and Pirh2 A190V were built from the structure of Pirh2 RING-H2 domain (PDB code: 2jrj). Interaction model of Pirh2–UbcH7 was built by superimposing the model of Pirh2 over C-cbl from C-cbl–UbcH7 complex (PDB code: 1fbv). Potential interactions and figures were generated using Pymol (www.pymol.org [25]).

## Results

### Validation

SIFT was originally validated upon previously published large-scale mutagenesis experiments [26]–[29], so we used an identical dataset to validate whether or not B-SIFT could call deleterious mutants at a rate similar to that of the original SIFT. The *E. coli* LacI repressor mutagenesis dataset contained 4004 mutations with experimentally measured phenotypes that SIFT used for validation [16]. SIFT was able to predict deleterious mutations in LacI at approximately 68% total prediction accuracy rate [27], [29]. We analyzed this same dataset with our implementation of B-SIFT and, using a Receiver-Operator Characteristic (ROC) curve plot, we show that B-SIFT and the original SIFT have almost identical true positive/false positive trade-off rates (Fig. 1B) for detection of deleterious mutations.

The goal of B-SIFT is to enable prediction of activating mutations in addition to deleterious mutations that SIFT already predicts, and so we sought out a large-scale mutagenesis dataset with experimentally verified phenotypes for use in validation of B-SIFT's utility. Ng and Henikoff used LacI, HIV-1 protease, and bacteriophage T4 lysozyme in validation of SIFT, but none of these three datasets contains information about activating or gain-of-function phenotypes [26]–[29]. To test B-SIFT's ability to predict activating mutations, we turned to the SWISS-PROT protein database, which contains literature-curated entries of experimentally determined phenotypes for directed mutagenesis experiments across a large number of proteins [7] (SWISS-PROT MUTAGEN field). We filtered the complete set of mutagenesis data from SWISS-PROT release 56 for single amino acid substitution mutations, which resulted in a dataset containing 20787 mutations. Mutations are described with a text description of the experimentally determined phenotype, but these descriptions do not adhere to any kind of specific format or controlled vocabulary. To simplify the analysis, we implemented a simple parsing algorithm to categorize each mutation description as deleterious, neutral, activating, or uncategorized. This categorization was done by looking for specific keywords within the first few words of the description, and since there is no controlled vocabulary the accuracy of this categorization approach was validated by random sampling and manual examination (see Methods).

**Figure 2:**
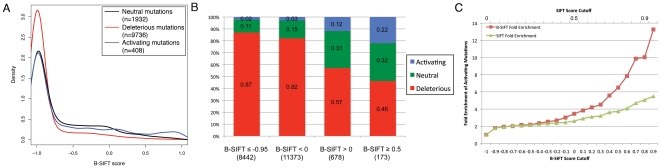
Validation of B-SIFT on protein mutation datasets. A. Distribution of B-SIFT scores for SWISS-PROT mutagenesis data. Density plots showing the distributions of B-SIFT scores for mutations in the SWISS-PROT mutagenesis dataset classified as deleterious (red curve), neutral (black), and activating (blue). Legend specifies the number of mutations classified under each functional category. B. Mutation composition of SWISS-PROT mutagenesis data. Each bar shows the percentage of the total mutations that meet the given B-SIFT cutoffs that are classified as either activating (blue), neutral (green), or deleterious (red). Values in parentheses show the total number of mutations that met each of the B-SIFT score thresholds. C. Fold enrichment of activating mutations with increasing score cutoffs. As B-SIFT score cutoff is increased, the percentage of activating mutations with B-SIFT scores greater than or equal to the cutoff increases as well (red line). A B-SIFT cutoff of −1 represents the complete dataset and each successive point is the fold enrichment over this baseline. In contrast, the green line shows a similar plot but using increasing SIFT cutoffs starting from 0. Although simply having a high SIFT score also results in enrichment of activating mutations, B-SIFT significantly improves the enrichment.

After the filtering by categorization, we were left with 14993 mutations categorized as either activating, neutral, or deleterious. These mutations were all analyzed by B-SIFT and filtered by information content of the SIFT alignment (see Methods), resulting in 12076 remaining mutations of which 408 (3.4%) are called activating and 9736 (80.6%) are deleterious. The majority of mutations scored near -1 in all three sets, but this is consistent with the fact that the majority of these mutations have SIFT scores close to zero (Figure S2). Even with the low-value peaks, however, distributions of B-SIFT scores for each of the three categories show enrichment at the expected B-SIFT values. For example, the deleterious mutations are enriched for low B-SIFT scores around -1, the neutral mutations have a score bump near 0 while the activating mutations have noticeably more mutations in the positive score range (Fig. 2A). This data can be seen in another form by examining the fraction of mutations with a given score cutoff that are classified as either deleterious, neutral, or activating (Fig. 2B). From this, we see that although only 3.4% of the total dataset is activating, 22% of the mutations with a B-SIFT score greater than 0.5 are activating (Fig. 2B) and in fact there is a consistent enrichment of activating mutations as B-SIFT scores increase (Fig. 2C). To show that the additional data used in the B-SIFT calculation improves performance, we also calculated the enrichment of activating mutations that would result from increasing SIFT scores alone. We also observe an enrichment of activating mutations for high SIFT scores, but B-SIFT performs substantially better (Fig. 2C). At a B-SIFT score cutoff of 0.5, we observe a 9% sensitivity towards identifying activating mutations but a 99% specificity, suggesting that we are able to identify only a subset of activating mutations but the majority of mutations are correctly classified as non-activating. There are many possible ways in which a mutant phenotype may be considered activating, but based on these results on diverse mutagenesis data we feel confident that B-SIFT is able to identify at least a subset of activating mutations that would not otherwise be identified by SIFT.

We further attempted to validate B-SIFT's ability to identify activating mutations through analysis of the Protein Mutant Database (PMD), a database of literature-curated protein mutants and phenotypes [30]. Although PMD contained a large number of mutations with phenotype descriptions and annotations, the interpretation of these descriptions proved to be more complex than the similar data contained in SWISS-PROT and the results were inconclusive. The primary obstacle towards proper utilization of these protein mutation databases as benchmarks for B-SIFT was the difficulty in assigning each phenotype as deleterious, neutral, or activating. We utilized an ad hoc method for doing initial categorization in both cases, but after extensive manual examination of the mutations classified as activating in both datasets, we found that the SWISS-PROT mutations are more likely to provide interpretable results. All 1170 mutations initially classified as activating in the two datasets (626 in SWISS-PROT and 544 in PMD) were examined by hand to call whether or not the mutation description was properly classified as activating. Although there is some subjectivity in this analysis, we found that ∼80% of SWISS-PROT and ∼64% of PMD mutations were correctly classified as activating, which typically required mention of increased enzymatic activity or substrate binding. Due to the difficulty in systematically categorizing activating mutants in PMD, our analysis focused instead on the SWISS-PROT mutants.

### Comparison with SNAP

SNAP (Screening for Non-Acceptable Polymorphisms) is a neural-network based computational tool trained on a large set of mutation data, including PMD data, that performs well in distinguishing neutral from non-neutral amino acid substitutions [6]. Since SNAP was trained on data that includes activating mutations, it specifically categorizes its predictions into two categories: neutral and non-neutral, where the authors intend non-neutral to include both deleterious and activating mutations. We applied SNAP to the same set of SWISS-PROT protein mutants mentioned above and examined the results on a large-scale.

SNAP outputs three values for each mutation: a binary call of neutral or non-neutral, a reliability index (RI), and an expected accuracy. The reliability index and expected accuracy are quality scores that are highly correlated, and so we only used the reliability index scores for quality thresholding. 14813 mutations across 4052 protein sequences received both a B-SIFT score and a SNAP prediction, and so we focused on these mutations for further analysis.

SNAP predicted only 1731 (11.3%) of these mutants to be neutral, so the great majority of mutations were predicted to be non-neutral. In order to investigate SNAP's ability to distinguish activating and neutral mutations, we calculated the percentage of mutations called neutral for each of the three categories at each reliability index (Figure S3). We found that neutral and activating mutations have a very similar distribution of SNAP calls until the reliability index cutoff is raised to 5 or higher, after which activating mutations are called non-neutral at a rate more similar to deleterious mutations. If we consider all predictions, we find that SNAP calls 74% of all activating mutations as non-neutral. However, among activating mutations with B-SIFT score greater than or equal to 0.5, SNAP calls only 26% as non-neutral, implying that B-SIFT is detecting a distinct subset of activating mutations.

### SNP Analysis

**Figure 3:**
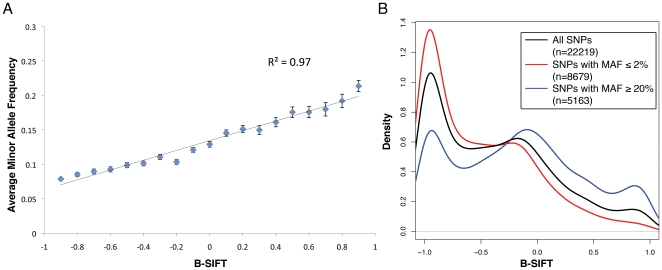
B-SIFT analysis of naturally occurring variations in dbSNP. A. Average minor allele frequency is correlated with B-SIFT score in dbSNP. Scatter plot and linear trendline showing that as B-SIFT score increases, the average minor allele frequency (MAF) for bi-allelic SNPs within each B-SIFT score range also increases, linear regression r^2^ = 0.97, error bars represent the standard error of the mean at each point. B. Distribution of B-SIFT scores in dbSNP. Density plots showing the distributions of B-SIFT scores for all bi-allelic polymorphisms in dbSNP (black curve), those with minor allele frequency (MAF) less than or equal to 2% (red), and those with MAF> = 20% (blue). The legend shows the number of SNPs included in each of the distribution curves.

Although the majority of our analysis is focused on mutations, we sought to ensure that B-SIFT is not simply identifying naturally occurring alleles in polymorphic positions. To do this, we leveraged the knowledge of natural human genetic variation in dbSNP to study the relationship between human population allele frequencies and functional prediction. We analyzed ∼32,000 missense SNPs from dbSNP for both B-SIFT score and allele frequency, calculating SIFT scores for both the reference allele and the variant to receive a B-SIFT value. These results were then filtered by information content of the alignment in the same way as the SWISS-PROT mutagenesis analysis to result in a list of ∼22,000 SNPs. Given that evolution will tend to select against mutations that provide a fitness disadvantage, we would believe that the “wild-type” or reference allele should be less likely to be deleterious than a less common polymorphic allele. However, we found that in some cases the reference allele is not the most common allele, and this can confound the B-SIFT results, and so we proceeded to calculate B-SIFT scores treating the more common allele as the “wild-type.” We then calculated the average minor allele frequency (MAF) for SNPs with varying B-SIFT cutoffs and observed a striking positive correlation (Fig. 3A, r^2^ = 0.97). In other words, residues that have been selected throughout the population to be primarily just a single allele (and therefore have a low minor allele frequency) are more likely to be deleterious when a different, less preferred allele is present. We would also expect that, in general, polymorphic positions that are tolerant of multiple high frequency alleles should be functional with either allele present. This is confirmed by showing B-SIFT score distribution for SNPs with different minor allele frequencies (Fig. 3B). SNPs with a low minor allele frequency (< = 2%, Fig. 3B, red line) are much more likely to be deleterious with that minor allele, whereas those that have high MAF (> = 20%, Fig. 3B, blue line) are much more likely to be tolerant. The distribution of all B-SIFT scores among available SNPs reveals a tri-modal distribution with peaks near −1, 0, and 1 (Fig. 3B, black line). These results show that it is possible for high B-SIFT scores to be a result of a common polymorphism, and so our mutation analyses have been filtered against known SNPs.

### Somatic Mutations in Cancer

The rapidly decreasing price and rising throughput of DNA sequencing has resulted in several efforts to identify somatic mutations in cancer in a comprehensive manner [8]–[14]. It is known that in many cases, the genetic event that drives tumorigenesis is a single or sequence of somatic mutations that results in a cancerous cell. Current cancer genome sequencing efforts are primarily focused on the protein-coding regions of the genome and so the majority of identified mutations are in the form of single-amino acid substitution changes. Numerous methods have been applied to the mutations discovered in these sequencing projects in an attempt to identify the causal mutations, but the majority of this analysis has been based upon methods that can only identify deleterious mutations [1]–[5].

**Figure 4:**
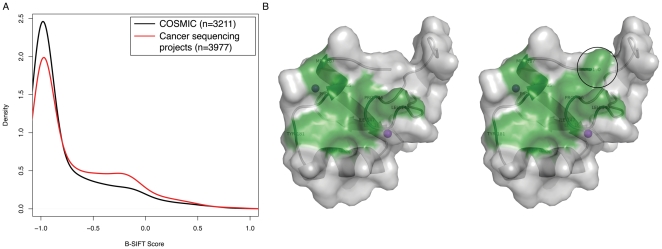
B-SIFT and structural analysis of potential activating cancer somatic mutations. A. Distribution of B-SIFT scores in cancer somatic mutation datasets. Density plots showing the distributions of B-SIFT scores for somatic missense mutations listed in COSMIC (black curve) [23], [24] and those found in large-scale cancer sequencing projects representing a large set of cancers including pancreatic, breast, colorectal cancers, lung adenocarcinoma, and glioblastoma (red) [8], [9], [11], [12], [14]. B. Model of Pirh2 interaction surface. Models of Pirh2 at the UbcH2 binding interface, green shading represents the hydrophobic surface important in the protein-protein interaction. The left model is for wild-type Pirh2 and the model on the right shows the increased hydrophobic surface that would result from the A190V mutation, the black circle highlights the change.

We have applied B-SIFT towards functional prediction of over 9000 mutations covering somatic alterations in multiple cancer types, including breast, colorectal, glioblastoma, pancreatic, and lung [8]–[14]. In order to ensure that none of our hits are actually high frequency polymorphisms, we first checked the list of somatic mutations against dbSNP and two other fully sequenced human genomes [31], [32]. The data can be separated into two sets; one set of mutations is extracted from the COSMIC database, which is filtered for mutations more likely to be causal, and the rest of the data consists of mutations identified from large-scale sequencing efforts comparing tumor samples to matched normal samples of the same individual [23], [24]. The COSMIC dataset is presumably enriched for functionally relevant mutations already whereas the large-scale somatic mutation discovery datasets should contain a fair number of “passenger” mutations that are functionally neutral. B-SIFT score distribution for each of the two sets of mutations confirms this hypothesis (Fig. 4A). A larger proportion of COSMIC mutations have very low B-SIFT scores, suggesting that many COSMIC mutations are functionally deleterious. On the other hand, the B-SIFT score distribution for somatic mutations discovered by large-scale sequencing projects has a noticeable bump near zero, suggesting that there is indeed a larger portion of these mutations that are not functionally relevant.

A small fraction of these somatic mutations have positive B-SIFT scores. Based on previous validation results, we speculate that this group of mutations may be enriched for activating mutations that have the potential to drive cancer. Table 1 shows a representative list of mutations with B-SIFT score>0.5 or with both a moderately high score (>0.2) and additional support from expression data (Gene Logic) showing that this gene may be overexpressed in cancer in the tissue that the mutation was found. The expression requirement attempts to find genes where additional protein function may be involved in cancer development or progression.

**Table 1:** Possible activating mutations found in cancer sequencing projects.

Refseq	Gene	Mutation	B-SIFT	Tissue*
NM_002208	ITGAE	V913I	0.92	Colorectal+
NM_014269	ADAM29	P31L	0.91	Colorectal
NM_015436	PIRH2	A190V	0.83	Pancreatic
NM_006218	PIK3CA	H1047L	0.82	Breast
NM_001039029	LRTM2	V320I	0.81	Colorectal
NM_014788	TRIM14	P207L	0.79	GBM+
NM_144773	GPR73L1	M165I	0.66	GBM
NM_007181	MAP4K1	A503S	0.66	Lung+
NM_015078	MCF2L2	R622H	0.65	Colorectal
NM_194251	GPR151	G68R	0.63	Pancreatic
NM_001523	HAS1	V521I	0.61	Pancreatic
NM_000059	BRCA2	P920S	0.60	CGA_GBM+
NM_005883	APC2	G2003S	0.53	Breast
NM_015199	ANKRD28	G651E	0.53	Breast
NM_002578	PAK3	P53T	0.52	Lung
NM_025132	WDR19	L214F	0.51	Colorectal
NM_133493	CD109	Q1007E	0.51	Colorectal
NM_030961	TRIM56	P356S	0.45	GBM+
NM_001569	IRAK1	C307F	0.43	Lung+
NM_003920	TIMELESS	Q1008E	0.40	Breast+
NM_001262	CDKN2C	M1I	0.33	Lung+
NM_005378	MYCN	L402F	0.33	Lung+
NM_020341	PAK7	P76T	0.32	Lung+
NM_020341	PAK7	T397K	0.31	Lung+
NM_001078	VCAM1	G395R	0.24	GBM+

* Tissue refers to the cancer tissue that this particular mutation was found in. Colorectal and breast are from Wood et al. [14], Pancreatic is from Jones et al. [11], GBM refers to glioblastoma data from Parsons et al. [12], CGA_GBM is glioblastoma data from The Cancer Genome Atlas publication [8], and lung refers to lung adenocarcinoma data from Ding et al. [9].

+in the Tissue column denotes that this gene is significantly overexpressed in cancer (p<0.01, t-test), compared to normal samples of the same tissue type (Gene Logic expression data, see Methods).

### Structural Analysis

To gain additional insights into this collection of somatic mutations in cancer, we evaluated whether some of these mutations would be consistent with functional activation based on protein structure analysis. We took the full set of B-SIFT results for somatic mutations found in high-throughput cancer sequencing datasets and filtered based on alignment quality, B-SIFT score, and available expression information (see Methods). The resulting mutations were then mapped to available protein structures (either exact structure or by homology) through queries to the Unison database [33]. A total of seventeen mutations could be mapped to a protein structure with a sequence identity of 50% or greater, which we then analyzed for their potential impact on protein function. We present below our arguments for two of these potentially activating mutations, with detailed analysis of a third example in Results S1 and Figures S4 and S5.

One of the somatic mutations with a high B-SIFT is H1047L in the gene phosphatidylinositol-3 kinase alpha isoform (PIK3CA), with a score of 0.82. PIK3CA is a well-known oncogene and H1047L is in a known cancer-associated mutation hotspot within the kinase domain [34]. This mutation has been shown to be an activating mutation both experimentally [35] and computationally [36]. Histidine-1047 is located close to the activation loop and the change from histidine to leucine results in loss of interactions with the activation loop making it more flexible. This increase in flexibility of the activation loop is presumed to result in increased substrate interaction thus leading to a gain of function. PIK3CA H1047L is a validation of B-SIFT's ability to identify potentially activating mutations.

The mutation A190V in the gene Pirh2 (p53-induced protein with RING-H2 domain) also gets a high B-SIFT score of 0.83. Pirh2 is an E3 Ubiquitin ligase and is known to negatively regulate levels of p53, a powerful tumor suppressor, in the cell [37]. Because Pirh2 promotes p53 degradation through ubiquitination, additional activity of Pirh2 will result in loss of p53 activity that will in turn result in cancer [38]. Alanine-190 is located at the C-terminal end of the central RING-H2 domain. RING-H2 domains mediate the interaction with the E2 enzyme while transferring Ubiquitin from the E2 enzyme to the substrate (in this case, p53). The interaction between the RING-H2 domain and E2 is known to be hydrophobically driven [39]. The RING-H2 domain of Pirh2 contains a shallow hydrophobic patch on its surface, a feature necessary to facilitate this interaction and shared by other E3 RING-H2 domains like C-cbl [39]. The structures of the three domains of Pirh2 were separately solved using NMR spectroscopy [40]. The structure of the RING-H2 domain has only been solved from residue 127 to 189, and so coordinates of Ala 190 were not available due to a flexible linker region between the RING-H2 domain and the C-terminal domain. We approached this analysis by building separate structural models of the Pirh2 RING-H2 domain with alanine and valine at position 190 using Modeler [41]. We then built an interaction model of Pirh2–UbcH7 using the coordinates of the C-cbl-UbcH7 complex. Figure 4B shows the hydrophobic patch on Pirh2 at the UbcH7 interface (green highlight), the increase in hydrophobicity when position 190 is mutated from alanine to valine is denoted by the larger hydrophobic patch (Fig. 4B). Based on previous interaction studies of RING and HECT E3 ligases with E2 enzymes [39], [42], [43], we hypothesize that this increase in hydrophobicity could result in an increase in binding affinity between the E3 and E2 proteins that would enhance p53 degradation.

## Discussion

We have presented evidence that the bi-directional SIFT algorithm is capable of finding a subset of mutations that are potentially functionally activating. This fills an important void in existing methods for functional analysis of mutations in that there are no current methods that have been established for identifying activating or gain-of-function mutations. B-SIFT is not only capable of filtering for activating mutations, but its accuracy in identifying deleterious mutations is consistent with that of the original SIFT algorithm.

There are some caveats to the study of activating mutations which are independent of the algorithm used, but do apply to B-SIFT. In particular, in many cases it is difficult to define exactly what “hyperactivity” means for a given protein. For example, in the DNase I data that we present, we define activity as DNA hydrolysis rate. However, there are many factors that can affect the rate of DNA hydrolysis, including DNA binding, actin binding, and the actual catalysis of the hydrolysis reaction [19], [44], [45]. In most cases, it appears that increasing DNA binding affinity improves the rate of DNA hydrolysis, but if the enzyme binds DNA too tightly then overall DNA hydrolysis can become inhibited by reducing the turnover of new DNA strands on an individual enzyme molecule.

Perhaps in a more familiar example, there are many examples of oncogenes in cancer that become tumorigenic as a result of hyperactivity. Many of these oncogenes are signaling molecules or receptors that become hyperactive or constitutively active which can result in uncontrolled cell proliferation [46]. There are many examples where this misregulated signaling is actually a result of a loss of function in a regulatory region of a signaling molecule [46]–[50]. In these cases, although it is hyperactivation of signaling that results in oncogenesis, it is in fact a loss-of-function mutation that results in this hyperactivity. For example, the L858R mutation in EGFR is a common mutation in cancer and is classified as an activating mutation, but its effect is a result of the mutation destabilizing the inactive conformation of the enzyme and causing it to fold into an active conformation even in the absence of ligand [51]. Similarly, it is hypothesized that the common V600E mutation in the BRAF kinase leads to excessive activation of the enzyme by mimicking phosphorylation and destabilizing its inactive conformation [52]. B-SIFT will fail to recognize most of these as being activating mutations, but it does in fact find many of these as deleterious mutations instead (EGFR L858R B-SIFT = −1, BRAF V600E B-SIFT = −1). Our analysis of COSMIC mutations and other cancer mutations did not find an enrichment for high B-SIFT scores in COSMIC (Fig. 4A), implying that the majority of characterized “activating” cancer mutations fall into this category of deleterious mutations that result in functional activation and are thus indistinguishable from other deleterious mutations by B-SIFT. The complexity involved in analysis of activating mutations is further demonstrated by the difficulty in categorizing mutations found in protein databases as deleterious, neutral, or activating. Without a controlled vocabulary or a clear definition of what constitutes an activating mutation, especially in the case of mutations with multiple known phenotypes, systematic identification will continue to be a challenge.

Our detailed structural analysis of cancer associated somatic mutations has found several examples of mutations that could contribute to cancer progression through different mechanisms, all with high B-SIFT scores. PIK3CA H1047L is an activating mutation in a well-studied gene with many known activating mutations that result in cancer [34], [53]. Pirh2 is also a known oncogene, but an activating mutation in this gene results in cancer indirectly by excessively degrading the p53 tumor suppressor.

It is apparent from our B-SIFT analysis that the systematic prediction of activating mutations is more complex than the analogous prediction of deleterious mutations. One confounding factor is that it seems as though the majority of possible mutations will in fact result in loss of function, and so the total sample size of activating mutations is significantly less. This is consistent with the SWISS-PROT mutagenesis dataset, in which only 3.4% of mutations appear to be gain of function mutations. Although these mutations are not an unbiased random sampling of all possible mutations, conventional wisdom is that it is much easier to disrupt protein function than to enhance it in some way, and the distribution of mutation descriptions supports this (∼80% deleterious). B-SIFT produces scores in a way consistent with the expectation that many more mutations will be deleterious than activating. In every dataset examined, the distribution of B-SIFT scores is shifted towards the negative end (Fig. 4A).

The inherent differences between activating and deleterious mutations are perhaps the greatest contributing factors towards the relative inaccuracy in prediction quality between the two mutation types. We find that the use of a B-SIFT cutoff allows for enriching a mutation dataset for activating mutations, but there continues to be a high rate of false negatives and false positives (Fig. 2B). False positives may result from alleles that are evolutionarily conserved and potentially provide a fitness advantage to the organism, but do not result in measurable optimized protein function. False negatives could be a result of the multiple sequence alignment that B-SIFT (and SIFT) relies upon being limited in its scope. In the case of activating mutations, if the mutant residue is not used by any of the homologues used in the B-SIFT alignment, then the algorithm is unlikely to score the mutation as activating. However, it is certainly possible that there exist activating mutations that are not otherwise seen in homologous protein sequences. On the other end of the spectrum, since it does seem as though the majority of mutations result in loss of function, it is likely that false positives result from the somewhat delicate nature of protein structure and function. Even though protein homologues may be functional with the mutated residue at the given position, even subtle differences in protein structure could result in vast differences in function as a result of the mutation. Although many caveats exist in the study of activating mutations, B-SIFT provides a starting point by finding mutations that would otherwise have been missed or indistinguishable from the deleterious mutations that comprise the majority of currently identified functional mutations.

Our analysis of large mutation datasets shows that B-SIFT is easily scalable in the way that SIFT is, and the distribution of B-SIFT scores can be used to discover high-level characteristics of the dataset. Furthermore, studies that are interested in finding activating mutations would find B-SIFT to be a useful tool in providing a first step for finding mutations most likely to be activating. There is still significant improvement that is possible in the field of detecting and characterizing activating mutations, but B-SIFT provides a valuable starting point for such analyses.

### Supplemental Data

Supplemental data are available for download at http://research-pub.gene.com/bsift/.

## Supporting Information

Results S1 (0.07 MB DOC)

Figure S1 DNase I activity for mutants with positive and negative B-SIFT scores. Each bar shows the mean relative specific activity (RSA) for DNase I mutants with positive B-SIFT scores (left bar), negative scores (right bar), or wild-type controls (middle). Error bars are the standard error of the mean for each dataset.
(0.73 MB TIF)

Figure S2 Distribution of Swiss-Prot mutant SIFT scores. SIFT scores of all Swiss-Prot mutants are shifted towards zero, which contributes to the large number of small B-SIFT scores among all mutation sets as shown in Figure 2A.
(0.49 MB PDF)

Figure S3 Percentage of Swiss-Prot mutations called as Neutral by SNAP, as separated by mutation category. Activating and neutral Swiss-Prot mutations show similar distributions of SNAP calls until higher Reliability Index cutoffs.
(0.73 MB TIF)

Figure S4 VCAM-1 gene expression in brain tissues. Boxplots of VCAM-1 expression show the distribution of expression values between cancerous and normal brain tissues. VCAM-1 is significantly overexpressed in cancer compared to normal in the brain.
(0.77 MB TIF)

Figure S5 VCAM-1 G395R-VLA4 interaction model. Cartoon representation of VCAM-1 domains 4 and 5 (orange) shown bound to VLA4 β1 subunit (translucent surface). G395R and D352 are shown as sticks. The MIDAS, ADMIDAS and LIMBS sites are shown in magenta, red, and green spheres respectively. Known and potential interactions are shown in blue and red dashed lines. The inset shows a close-up view of these interactions.
(10.66 MB PDF)
