# Supplementary material for: Bi-Directional SIFT Predicts a Subset of Activating Mutations
Source: PLoS One. 2009 Dec 14;4(12):e8311. doi: 10.1371/journal.pone.0008311 (PMC2788704; doi:10.1371/journal.pone.0008311)
Supplement: Results S1 — (0.07 MB DOC) [file pone.0008311.s001.doc]

**SUPPLEMENTAL RESULTS**

**DNase I hyperactivity analysis**

Following the example set by the individual protein mutagenesis experiments, we attempted to predict the functional impact of all possible single amino acid substitution mutations in another protein. Here we chose human deoxyribonuclease I (DNase I), which belongs to a family of human DNA-cleaving endonucleases[1,2]. In particular, DNase I has been very well studied by functional mutagenesis studies[3-8]. Recombinant human DNase I (Pulmozyme® dornase alfa) has also been used clinically where it is aerosolized into the airways for treatment of pulmonary disease in patients with cystic fibrosis[9,10]. Our ability to predict mutations that result in greater enzymatic activity could in principal lead to a drug that has greater therapeutic potency. DNase I mutants covering 160 unique single amino acid substitutions were assayed with a colorimetric assay to determine DNA hydrolysis activity in a quantitative manner[11]. These enzymatic activity measurements were then compared against a comprehensive B-SIFT analysis of human DNase I.

Human DNase I is a secreted enzyme with a mature length of 260 amino acids[9] and thus has a total of 4940 total possible mutations that could encode the full-length protein. Out of the 260 residues, 88 had a wild-type SIFT score less than 1, meaning that these are the only positions that could potentially result in a positive B-SIFT score. Within these 88 positions, B-SIFT analysis identified 320 mutations with a positive B-SIFT score, representing 6.5% of all mutations. We measured phenotypes for mutants covering 77 unique mutations in 14 of these 88 positions, the rest of the dataset covers 83 unique mutations in 32 different positions. In total, we studied 13 mutations across 8 amino acid positions with a positive B-SIFT score.

The methyl green assay for DNase activity provides a quantitative measurement that can be normalized to yield a relative specific activity (RSA) score for comparison of all mutants and experiments. All mutants and wild-type controls were assayed for DNase I concentration using an ELISA and DNA hydrolysis activity using the methyl green assay. We first sought to investigate whether or not mutants that had higher B-SIFT scores would show a greater amount of enzymatic activity. The complete set of activity measurements was divided into three categories representing mutants with positive B-SIFT scores, negative scores, and the wild-type controls. The distribution of RSA measurements for each of these sets of mutants shows that mutants that have high B-SIFT scores do tend to have greater activity (Figure S1, Mann-Whitney p-value = 0.04 pos vs. neg). These results confirm that B-SIFT is able to successfully separate activating mutants from functionally inactive and deleterious mutants in a quantitative manner and could thus be a useful tool for future protein engineering efforts.

**VCAM-1 Structural Analysis**

The mutation G395R in the cell adhesion protein VCAM-1 (vascular cell adhesion molecule 1) received a modest B-SIFT score of 0.24, but additional expression data suggests that increased protein activity is associated with cancer (Figure S4). Cell adhesion molecules (CAMs) play a key role in mediating cell-cell interactions by binding to integrin receptors expressed on the surface of adjacent cells. VCAM-1 is a member of the immunoglobulin (Ig) superfamily and has a structure that contains seven extracellular Ig-like domains. It binds to cells expressing the integrin α4β1 (encoded by VLA4, very late antigen 4) and mediates leucocyte-endothelial cell adhesion and signal transduction. Domains 1 and 4 of VCAM-1 are known to be involved in receptor binding[12]. Two sequence motifs, IDSP and KLEK, have been shown to participate in receptor binding in domain 1[13] and both of the motifs are conserved in the homologous domain 4. The positively charged residue in the IDSP motif (typically aspartic acid, but some ligands have a glutamic acid in its place) is common to other integrin ligands and is the key residue involved in integrin recognition. In our case, the G395R mutation falls right after the KLEK sequence motif in domain 4. Based on the proximity of the G395R mutation to the binding site, we investigated the effect of the mutation on receptor binding. As the structures of VCAM-1 domain 4 and VLA4 were not available, our first goal was to a build a homology model of the ligand-receptor complex (Figure S5). We built a homology model of domains 4 and 5 of VCAM-1 based the solved structure of domains 1 and 2 respectively. VLA4, like other integrins, is a heterodimer of the α and β subunits. The structure of a related integrin αVβ3 was solved in complex with a peptide ligand RGD that mimics binding sites from integrin ligands[14]. The structure reveals that the key aspartic acid from the peptide ligand and the site corresponding to VCAM-1 G395R primarily interact with the A domain of the β subunit. We built a homology model of VLA4 β1 subunit using the β3 subunit structure including three divalent cations at the MIDAS (the metal-ion independent adhesion site), ADMIDAS (adjacent to MIDAS) and LIMBS (ligand-associated metal-binding site) domains that are known to regulate integrin-ligand interactions. We then used the template structure to dock VCAM-1 domain 4 to VLA4 by superposing the integrin binding aspartic acid residues. In the β1 – VCAM-1 complex model, the mutation from glycine to arginine does not break any critical structural contacts within VCAM-1 but falls on a solvent exposed edge of the Ig-like domain beta sheet. The arginine side chain is positioned to interact with the ADMIDAS site. The arginine side chain is within interacting distance from three aspartic acid residues (D158, D159, D280) that coordinate with the ADMIDAS cation and are conserved in β3, potentially forming a salt bridge with acidic residues or hydrogen bonds with the backbone carbonyl oxygens at the ADMIDAS site. We hypothesize that the G395R mutation stabilizes the ligand-receptor complex due to these interactions and hence is an activating mutation.

**SUPPLEMENTAL DATA**

Additional supplemental data is available for download at:

http://research-pub.gene.com/bsift/

**SUPPLEMENTAL FIGURES LEGENDS**

**Figure S1. DNase I activity for mutants with positive and negative B-SIFT scores**

Each bar shows the mean relative specific activity (RSA) for DNase I mutants with positive B-SIFT scores (left bar), negative scores (right bar), or wild-type controls (middle). Error bars are the standard error of the mean for each dataset.

**Figure S2. Distribution of Swiss-Prot mutant SIFT scores**

SIFT scores of all Swiss-Prot mutants are shifted towards zero, which contributes to the large number of small B-SIFT scores among all mutation sets as shown in Figure 2A.

**Figure S3. Percentage of Swiss-Prot mutations called as Neutral by SNAP, as separated by mutation category**

Activating and neutral Swiss-Prot mutations show similar distributions of SNAP calls until higher Reliability Index cutoffs.

**Figure S4. VCAM-1 gene expression in brain tissues**

Boxplots of VCAM-1 expression show the distribution of expression values between cancerous and normal brain tissues. VCAM-1 is significantly overexpressed in cancer compared to normal in the brain.

**Figure S5. VCAM-1 G395R-VLA4 interaction model**
Cartoon representation of VCAM-1 domains 4 and 5 (orange) shown bound to VLA4 β1 subunit (translucent surface). G395R and D352 are shown as sticks. The MIDAS, ADMIDAS and LIMBS sites are shown in magenta, red, and green spheres respectively. Known and potential interactions are shown in blue and red dashed lines. The inset shows a close-up view of these interactions.

**REFERENCES**

1. Baranovskii AG, Buneva VN, Nevinsky GA (2004) Human deoxyribonucleases. Biochemistry (Mosc) 69: 587-601.

2. Lazarus RA (2002) Human Deoxyribonucleases. In: Creighton TE, editor. Wiley Encyclopedia of Molecular Medicine. New York: John Wiley and Sons. pp. 1025-1028.

3. Pan CQ, Dodge TH, Baker DL, Prince WS, Sinicropi DV, et al. (1998) Improved potency of hyperactive and actin-resistant human DNase I variants for treatment of cystic fibrosis and systemic lupus erythematosus. J Biol Chem 273: 18374-18381.

4. Pan CQ, Lazarus RA (1997) Engineering hyperactive variants of human deoxyribonuclease I by altering its functional mechanism. Biochemistry 36: 6624-6632.

5. Pan CQ, Lazarus RA (1998) Hyperactivity of human DNase I variants. Dependence on the number of positively charged residues and concentration, length, and environment of DNA. J Biol Chem 273: 11701-11708.

6. Pan CQ, Lazarus RA (1999) Ca2+-dependent activity of human DNase I and its hyperactive variants. Protein Sci 8: 1780-1788.

7. Pan CQ, Ulmer JS, Herzka A, Lazarus RA (1998) Mutational analysis of human DNase I at the DNA binding interface: implications for DNA recognition, catalysis, and metal ion dependence. Protein Sci 7: 628-636.

8. Ulmer JS, Herzka A, Toy KJ, Baker DL, Dodge AH, et al. (1996) Engineering actin-resistant human DNase I for treatment of cystic fibrosis. Proc Natl Acad Sci U S A 93: 8225-8229.

9. Lazarus RA, Wagener JS (2007) Recombinant Human Deoxyribonuclease I. In: Crommelin DJA, Sindelar RD, Meibohm B, editors. Pharmaceutical Biotechnology: Fundamental and applications. 3rd ed. New York: Informa Healthcare. pp. 387-398

10. Suri R (2005) The use of human deoxyribonuclease (rhDNase) in the management of cystic fibrosis. BioDrugs 19: 135-144.

11. Sinicropi D, Baker DL, Prince WS, Shiffer K, Shak S (1994) Colorimetric determination of DNase I activity with a DNA-methyl green substrate. Anal Biochem 222: 351-358.

12. Osborn L, Vassallo C, Benjamin CD (1992) Activated endothelium binds lymphocytes through a novel binding site in the alternately spliced domain of vascular cell adhesion molecule-1. J Exp Med 176: 99-107.

13. Clements JM, Newham P, Shepherd M, Gilbert R, Dudgeon TJ, et al. (1994) Identification of a key integrin-binding sequence in VCAM-1 homologous to the LDV active site in fibronectin. J Cell Sci 107 ( Pt 8): 2127-2135.

14. Xiong JP, Stehle T, Diefenbach B, Zhang R, Dunker R, et al. (2001) Crystal structure of the extracellular segment of integrin alpha Vbeta3. Science 294: 339-345.
