# Supplementary figures and images for: Bi-Directional SIFT Predicts a Subset of Activating Mutations
Source: PLoS One. 2009 Dec 14;4(12):e8311. doi: 10.1371/journal.pone.0008311 (PMC2788704; doi:10.1371/journal.pone.0008311)

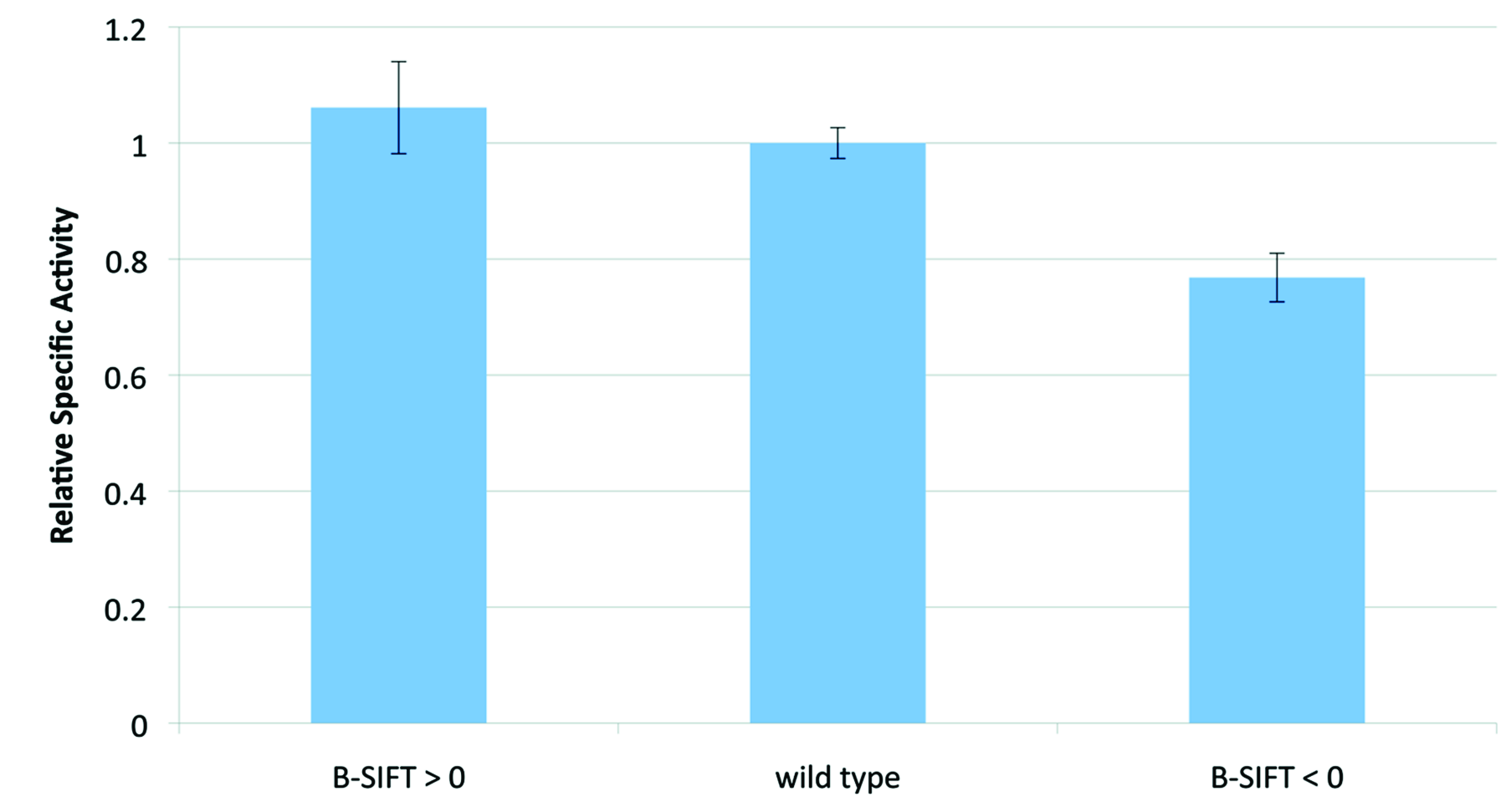

Supplement: Figure S1 — DNase I activity for mutants with positive and negative B-SIFT scores. Each bar shows the mean relative specific activity (RSA) for DNase I mutants with positive B-SIFT scores (left bar), negative scores (right bar), or wild-type controls (middle). Error bars are the standard error of the mean for each dataset. (0.73 MB TIF) [file pone.0008311.s002.tif]

**Distribution of Swiss-Prot mutant SIFT scores**

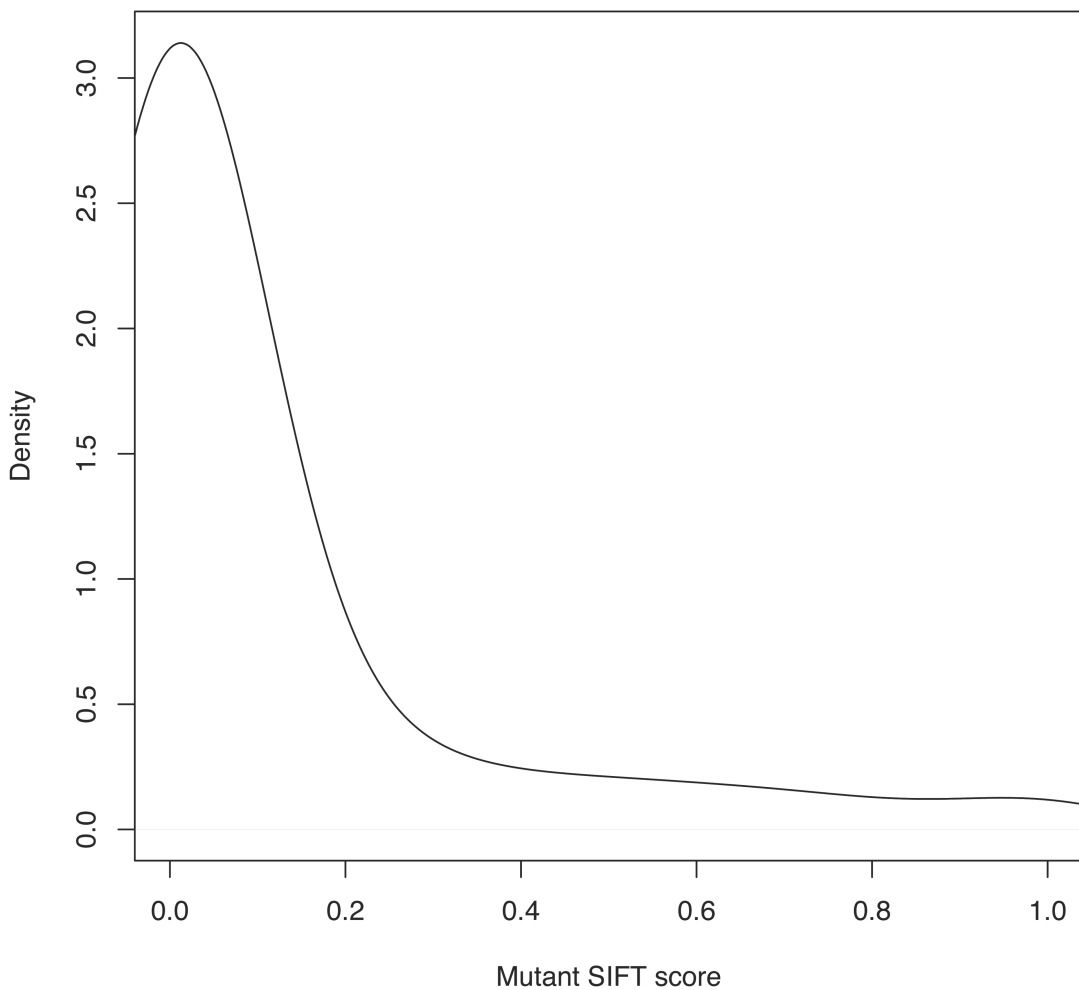

Supplement: Figure S2 — Distribution of Swiss-Prot mutant SIFT scores. SIFT scores of all Swiss-Prot mutants are shifted towards zero, which contributes to the large number of small B-SIFT scores among all mutation sets as shown in Figure 2A. (0.49 MB PDF) [file pone.0008311.s003.pdf]

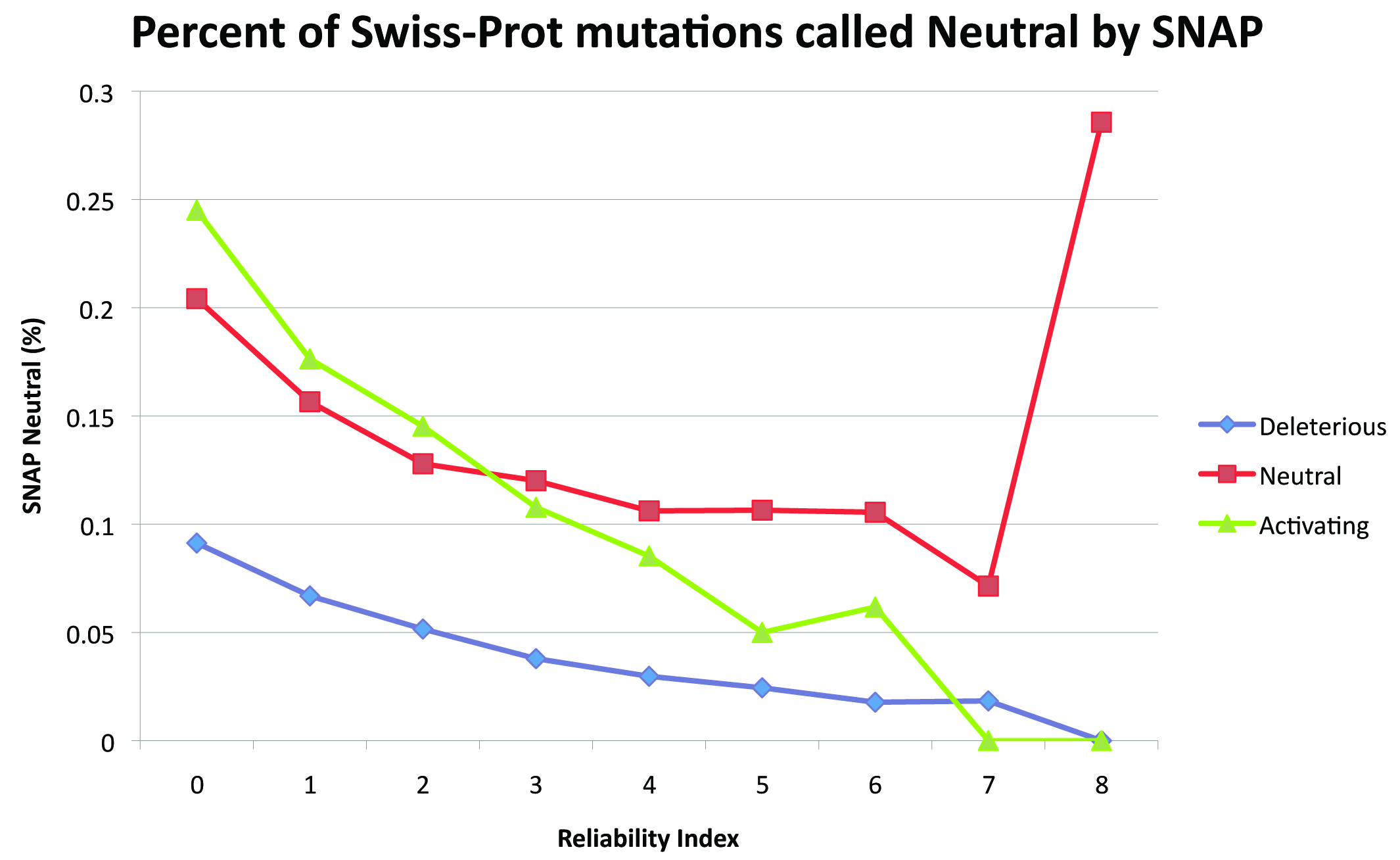

Supplement: Figure S3 — Percentage of Swiss-Prot mutations called as Neutral by SNAP, as separated by mutation category. Activating and neutral Swiss-Prot mutations show similar distributions of SNAP calls until higher Reliability Index cutoffs. (0.73 MB TIF) [file pone.0008311.s004.tif]

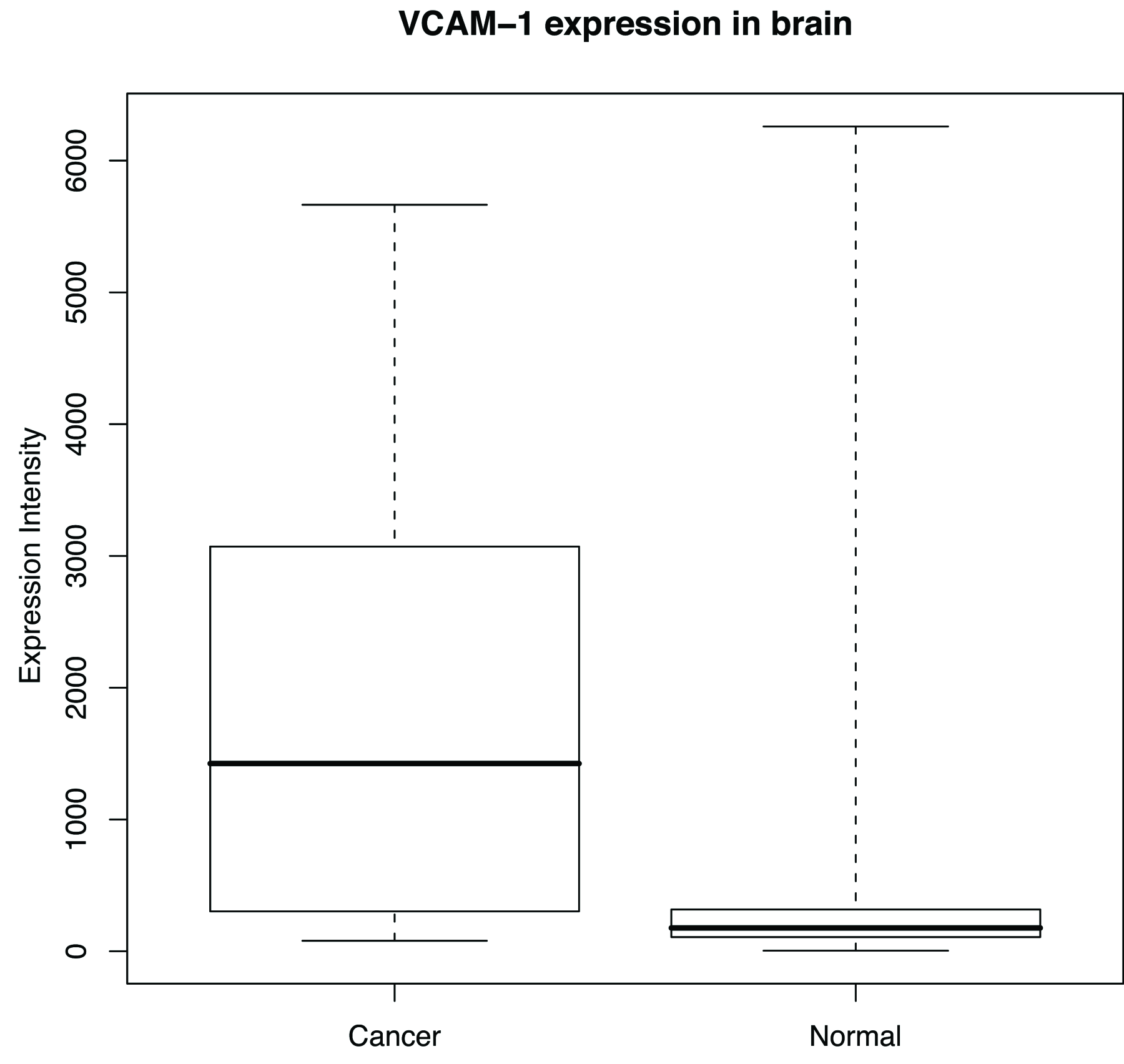

Supplement: Figure S4 — VCAM-1 gene expression in brain tissues. Boxplots of VCAM-1 expression show the distribution of expression values between cancerous and normal brain tissues. VCAM-1 is significantly overexpressed in cancer compared to normal in the brain. (0.77 MB TIF) [file pone.0008311.s005.tif]

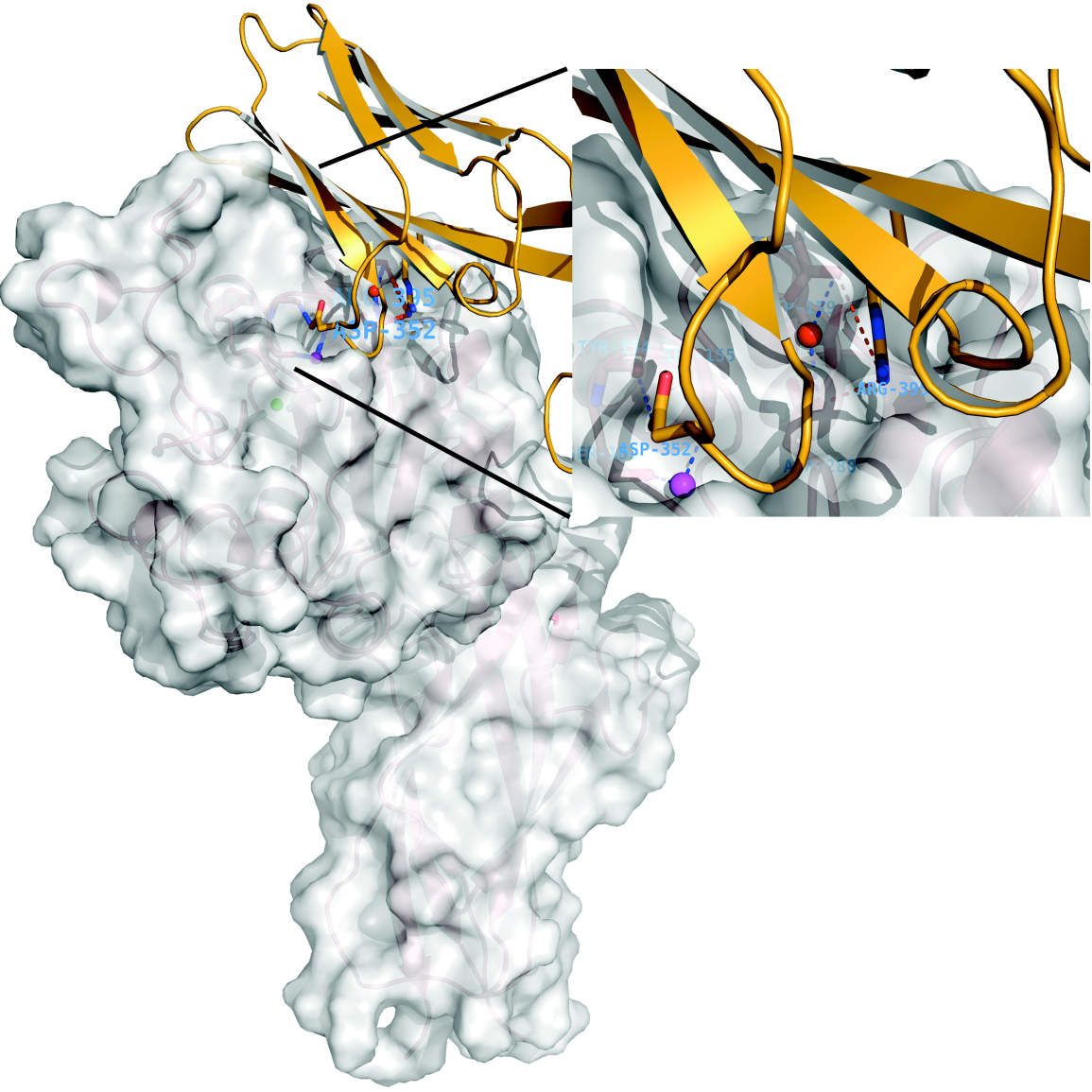

Supplement: Figure S5 — VCAM-1 G395R-VLA4 interaction model. Cartoon representation of VCAM-1 domains 4 and 5 (orange) shown bound to VLA4 β1 subunit (translucent surface). G395R and D352 are shown as sticks. The MIDAS, ADMIDAS and LIMBS sites are shown in magenta, red, and green spheres respectively. Known and potential interactions are shown in blue and red dashed lines. The inset shows a close-up view of these interactions. (10.66 MB PDF) [file pone.0008311.s006.pdf]
